# Enhancement of Lycopene Biosynthesis Using Self-Assembled Multi-Enzymic Protein Cages

**DOI:** 10.3390/microorganisms13040747

**Published:** 2025-03-26

**Authors:** Yulong Zhou, Yonghua Yao, Furong Zhang, Ning Yu, Binqiang Wang, Bing Tian

**Affiliations:** 1 Key Laboratory for Green Processing of Chemical Engineering of Xinjiang Bingtuan, School of Chemistry and Chemical Engineering, Shihezi University, Shihezi 832003, China; zhouyulong27jing@163.com (Y.Z.); yyh1458526019@163.com (Y.Y.); 2 Institute of Biophysics, College of Life Sciences, Zhejiang University, Hangzhou 310058, China; zhangfr@zju.edu.cn (F.Z.); yn2388690053@163.com (N.Y.)

**Keywords:** carboxysome, enzyme immobilization, IPP synthetic nanocage, lycopene production

## Abstract

Constructions of self-assembled protein nanocages for enzyme immobilization and cargo transport are very promising in biotechnology fields such as natural product biosynthesis. Here, we present an engineered isopentenyl pyrophosphate (IPP) synthetic nanocage with multiple enzymes for lycopene production in bacteria. The enzymes involved in IPP biosynthesis (ScCK, AtIPK, and MxanIDI) were assembled onto the exterior of an engineered protein cage based on α-carboxysome. The IPP synthetic nanocage was co-expressed with CrtE/CrtB/CrtI in *Escherichia coli*. This approach increased the metabolic flux and resulted in a 1.7-fold increase in lycopene production in the engineered *E*. *coli* compared with the control strain. The results provide insights into the immobilization and assembling of IPP biosynthetic enzymes in protein nanocages, which serve as a powerful tool for achieving efficient synthesis of lycopene.

## 1. Introduction

Cell compartmentalization spatially segregates various cellular functions within the cell into distinct compartments, providing a structural framework for the regulation and optimization of intracellular metabolic functions and efficiencies. This organization enables the simultaneous occurrence of diverse or cascading complex biochemical reactions without interference [1]. Cell compartmentalization offers numerous advantages, such as increasing the local concentrations of enzymes and substrates to improve catalytic efficiency, reducing the accumulation of toxic intermediates to minimize cytotoxic effects, and eliminating interference from competing pathways [2,3]. In recent years, the strategy of cellular compartmentalization has played a crucial role in the development of microbial cell factories for natural product synthesis.

Prokaryotes possess intricate proteinaceous cellular structures known as bacterial microcompartments (BMCs) [4], which are widely distributed across various bacteria and exhibit functionalities akin to eukaryotic organelles [5,6]. Some BMCs can be formed through the self-assembly of a series of homologous shell proteins, encapsulating enzymes associated with specific metabolic pathways [3,7]. Their remarkable potential in the design and fabrication of novel protein nanoreactors stems from intrinsic properties such as self-assembly, selective permeability, enzyme encapsulation efficiency, and high biocompatibility [8,9]. For instance, BMCs were used for the biosynthesis of ethanol in *Escherichia coli* [10]. Among these proteinaceous BMCs, carboxysomes are found in cyanobacteria and certain chemolithoautotrophic organisms that function as CO_2_ fixation mechanisms, playing a crucial role in the global carbon cycle [11]. The carboxysome encapsulates carbonic anhydrase (CA) and the primary carbon dioxide-fixing enzyme ribulose-1,5-bisphosphate carboxylase/oxygenase (RuBisCO) within a protein shell. This protein shell facilitates the selective entry of HCO_3_^−^, which is converted into CO_2_ by the CA in proximity to RuBisCO. Subsequently, RubisCO fixes the locally generated CO_2_ onto ribulose-1,5-bisphosphate (RuBP) molecules, yielding two molecules of 3-phosphoglycerate (3PGA) [12]. Concurrently, the protein shell restricts the passage of CO_2_, resulting in its efficient accumulation within the shell, thereby enhancing RuBisCO carboxylation and reducing photorespiration [13]. Based on the phylogeny of RuBisCO, the types of carboxysomes are usually classified into α-carboxysome found in marine cyanobacteria and β-carboxysome, which are mainly present in freshwater and estuarine cyanobacteria [14]. The assembly of β-carboxysomes occurs in an inward–out manner, necessitating the gradual formation of the RuBisCO core before the assembly of the shell [15,16]. In contrast, α-carboxysomes utilize strategies where the shell is formed first or where RuBisCO and shell proteins are assembled simultaneously [17,18]. Due to the inherent compartmentalization of α-carboxysomes, leveraging their protein shells to integrate diverse metabolic pathways facilitates the construction of innovative biological nanoreactors. Previous studies have demonstrated the potential of utilizing the α-carboxysome shell to encapsulate oxygen-sensitive hydrogenases for the development of hydrogen-producing nanoreactors [9]. However, despite the significant potential of carboxysome shells in various biotechnological applications, the complexity of their components presents challenges. Simplifying the carboxysome shell architecture and achieving tunable and efficient enzyme loading strategies are critical issues for the rational design and engineering of carboxysome-based nanostructures. Recently, engineered protein nanocages based on an α-carboxysome shell containing specific cargo loading sites using the SpyTag/SpyCatcher system were constructed and exhibited potential in enzyme immobilization and catalysis [19], which shows application potential in the field of natural product synthesis.

Terpenoids are widely distributed secondary metabolites in bacteria, plant, archaea, and fungi, among others [20,21], encompassing over 50,000 known types characterized by their diverse molecular structures and biological functions [22,23,24]. Many tetraterpenes (carotenoids) such as lycopene and β-carotene possess significant application value in the food and pharmaceutical industries [25,26]. There are two primary methods for obtaining tetraterpenes—biosynthesis and chemical synthesis [27]. Microbial biosynthesis of lycopene represents a sustainable and environmentally friendly production strategy. Consequently, the development of efficient microbial cell factories for lycopene synthesis has emerged as a significant research focus for sustainable production of the product. In bacteria, the lycopene synthesis pathway usually involves multiple enzymes using isopentenyl pyrophosphate (IPP) as C5 precursor substrate [28]. IPP and its allylic isomer, dimethylallyl pyrophosphate (DMAPP), serve as precursors in terpenoid biosynthesis. Geranylgeranyl diphosphate synthase (CrtE) catalyzes the head-to-tail condensation of these C5 building blocks to yield isoprenoid precursors, including geranyl diphosphate (GPP, C10), farnesyl diphosphate (FPP, C15), and geranylgeranyl diphosphate (GGPP, C20). The subsequent enzymes CrtB and CrtI are responsible for the conversion of GGPP to produce phytoene and then lycopene [29,30]. Moreover, IPP can be produced through the isoprenoid alcohol pathway [31,32,33,34], which is an artificial designed enzyme cascade involving choline kinase from *Saccharomyces cerevisiae* (ScCK) and isopentenyl phosphate kinase from *Arabidopsis thaliana* (AtIPK) using exogenous isoprenol as the substrate [31,32,33,34]. In recent years, with the rapid advancements in synthetic biology and metabolic engineering, synthesis of lycopene has been achieved in model microorganisms such as *E. coli* and yeast [35,36,37]. However, enzymes, metabolites, and cofactors associated with lycopene synthesis might disperse within a cell and are limited by local concentration of the enzymes, which impacts the efficiency of product synthesis [38,39,40]. The strategy of cellular compartmentalization employs organelles or subcellular structures for the reconstruction of terpenoid metabolic pathways in microbial cell factories will effectively address the aforementioned challenges. Previous studies have indicated that co-localizing the cascade enzymes involved in the biosynthesis of lycopene within the protein cage Mi3 can enhance lycopene production [41]. The Mi3 is an engineered dodecahedral protein cage derived from the aldolase of the hyperthermophilic bacterium *Thermotoga maritima*, comprising 60 identical subunits [42,43].

In this study, we developed an IPP-synthetic protein nanocage based on α-carboxysome of the marine bacterium *Prochlorococcus marinus* MED4. We co-immobilized the key enzymes of IPP synthesis (ScCK, AtIPK and MxanIDI) within the protein nanocage, which were co-expressed with CrtE, CrtB, and CrtI in *Escherichia coli* to enhance the production of lycopene. The function of the IPP-synthetic protein nanocage for lycopene synthesis was evaluated.

## 2. Materials and Methods

### 2.1. Materials

PCR primers used are listed as shown in Supplementary Table S1. PCR primers utilized in this study were synthesized from Tsingke Biotechnology (Beijing, China). The PCR was conducted using Q5 High-Fidelity DNA Polymerase (Tsingke, Beijing, China). PCR fragments were purified using gel extraction kits or DNA purification kits sourced from Omega (Shanghai, China). Homologous endonucleases were obtained from Abclonal (Wuhan, China). The plasmid extraction kits were supplied by Omega (Shanghai, China). All antibiotics were purchased from BBI (Shanghai, China). Lycopene and IPP were products of Sigma (St. Louis, MO, USA). DNA sequencing was performed at Tsingke Biotechnology (Beijing, China). *Escherichia coli* strains DH5α and BL21 (DE3) were acquired for plasmid construction and recombinant protein overexpression, respectively. The Luria–Bertani (LB) medium used for cloning and expression contained tryptone (10 g/L), yeast extract (5 g/L), and sodium chloride (10 g/L). The fermentation medium for lycopene production in shake flasks consisted of peptone (15 g/L), yeast extract (12 g/L), NaH_2_PO_4_ (3 g/L), K_2_HPO_4_ (7 g/L), NaCl (2.5 g/L), Tween-80 (5 g/L), and glycerol (10 g/L).

### 2.2. Plasmid Construction

For protein cage shell construction, the genes encoding carboxysome shell (CB) proteins from *Prochlorococcus marinus* MED4 were synthesized by Tsingke Biotechnology (Beijing, China). A plasmid containing SpyCatcher and CBs (pP15A-CBs-SC) was constructed following the previously described method [19]. The genes encoding *CrtE*, *CrtB*, and *CrtI* were cloned from *D. radiopugnans* and subsequently ligated into the pET28a plasmid.

For the cargos loading on the protein cage shell, IDI genes were amplified from *Deinococcus radiodurans* (DR), *Deinococcus radiopugnans* (DRP), and *Myxococcus xanthus* (MXAN), and subsequently cloned into PCDF plasmid. The genes encoding *ScCK*, *AtIPK*, and their fused genes for ST attachment to C-termini (ScCK-ST, AtIPK-ST), as well as *IDI-ST*, were synthesized by Tsingke Biotechnology (Beijing, China). These genes were subsequently cloned into the PCDF plasmid. The primer sequences are listed in Supplementary Table S1. The plasmids are listed in Supplementary Table S2. The genes are listed in Supplementary Table S4.

### 2.3. Expression and Assembly of Carboxysome Nanocage Scaffold

#### 2.3.1. Expression and Assembly of mCherry-Labeled Carboxysome Shell-SC and mGFP-ST Cargo Protein

The plasmids containing mCherry-labeled carboxysome shell-SC (CBs-mCherry-SC) protein cage or the ST-linked cargo protein mGFP (mGFP-ST), as listed in Supplementary Table S2, were co-expressed into *E. coli* strain ly001 (Supplementary Table S3), and we obtained the *E. coli* strain CBs1. A strain expressing CBs-mcherry-SC and mGFP was used as the control strain (CBs0). The bacterial strains were inoculated into LB medium supplemented with 34 μg mL^−1^ chloramphenicol and 40 μg mL^−1^ streptomycin, followed by incubation at 37 °C. When the optical density at 600 nm (OD_600_) reached 0.6, isopropyl β-D-1-thiogalactopyranoside (IPTG) was added to a final concentration of 0.25 mM to induce mGFP-ST cargo protein expression. After 3 h of IPTG induction, cumate was added at a final concentration of 50 mg L^−1^ to initiate shell protein expression and assembly. The cultures were then transferred to a shaker and incubated at 25 °C for 16 h. The strains are listed in Supplementary Table S3.

#### 2.3.2. Construction of Carboxysome Nanocages for Immobilization of IPP Synthetic Enzymes

The E. coli CBIUP1 strain with co-expressed CBs-SC and ST-linked IPP biosynthetic enzymes (Supplementary Table S3) was inoculated into LB medium containing 34 μg mL^−1^ chloramphenicol, 40 μg mL^−1^ streptomycin, and 50 μg mL^−1^ kanamycin, followed by incubation at 37 °C. Upon reaching OD_600_ = 0.6, IPTG was added to a final concentration of 0.4 mM to induce the expression of IPP biosynthetic enzymes (ScCK, AtIPK, IDI) and lycopene biosynthetic enzymes (CrtE/B/I). After 3 h of IPTG induction, cumate (50 mg L^−1^) was added to trigger shell protein expression and assembly. The cultures were subsequently incubated at 25 °C with continuous shaking for 48 h. Finally, bacterial cells were harvested via centrifugation for analysis.

### 2.4. Fluorescence Microscopy

Fluorescence imaging was conducted using a Nikon ECLIPSE Ti2-U inverted fluorescence microscope (Nikon Corporation, Tokyo, Japan). Green fluorescence of mGFP was detected at an excitation wavelength of 488 nm and an emission wavelength of 500–530 nm; the mCherry (red fluorescence) was detected at an excitation wavelength of 514 nm and an emission wavelength of 585–635 nm.

### 2.5. Transmission Electron Microscopy (TEM)

In the preparation of samples for TEM, *E. coli* cultures (CBs0 and CBs1) were grown in LB medium until the optical density at 600 nm (OD_600_) reached 0.6–0.8. The cultures were harvested by centrifugation at 4000× *g* for 10 min and washed three times with PBS. The *E. coli* pellet was resuspended in a 2.5% glutaraldehyde solution (prepared in PBS) and fixed overnight at 4 °C. The fixative was discarded, and the samples were rinsed three times with 0.1 M phosphate buffer, pH 7.0, for 15 min each. Subsequently, the samples were post-fixed in 1% osmium tetroxide for 1–2 h. Dehydration was performed using a series of ethanol solutions at room temperature, followed by overnight infiltration with pure embedding medium. Ultrathin sections of 70–90 nm were obtained using a LEICA EM UC7 ultramicrotome (Leica, Wetzlar, Germany). The sections were stained with lead citrate and a saturated solution of uranyl acetate in 50% ethanol for 5–10 min. Finally, the samples were examined using a Hitachi H-7650 transmission electron microscope (Hitachi, Tokyo, Japan).

### 2.6. Carotenoid Extraction

Bacterial cell culture was centrifuged to collected cell pellet, which was added with pre-chilled extraction solvent (acetone/methanol, *v*:*v* = 7:2) and agitate at 30 °C for 20 min. Subsequently, the mixture was centrifuged at 7000 rpm for 10 min and the obtained supernatant was transferred to a glass container. The pellet was extracted again with the solvent for 30 min, followed by another centrifugation at 7000 rpm. The supernatant was combined with the initial supernatant and freeze-dried.

### 2.7. Carotenoid Analysis Using HPLC

*Carotenoid* was analyzed using a Waters 2695 liquid chromatography (Waters, Milford, MA, USA) system equipped with a 2998 ultraviolet detector (Waters). The mobile phase consists of acetonitrile, methanol, and isopropanol in a ratio of 80:15:5, with a flow rate of 1 mL/min. The chromatographic column used was an XBridge-C18 (Waters) (5 μm, 4.6 × 250 mm). The detection wavelength was set at 472 nm, and the column temperature was maintained at 40 °C. A 10 μL sample dissolved in acetone was injected for HPLC assay.

### 2.8. Statistics Analysis

Statistical parameters, including the definition and the number of experimental repetitions (*n*) and their deviations, have been reported in the figures and corresponding legends. Data are presented as mean ± standard deviation. Statistical analyses were conducted using GraphPad Prism version 10.1.2.

## 3. Results

### 3.1. Construction of IPP Synthetic Multi-Enzyme Nanocage Using α-Carboxysome Shell Assembly

**Figure 1:**
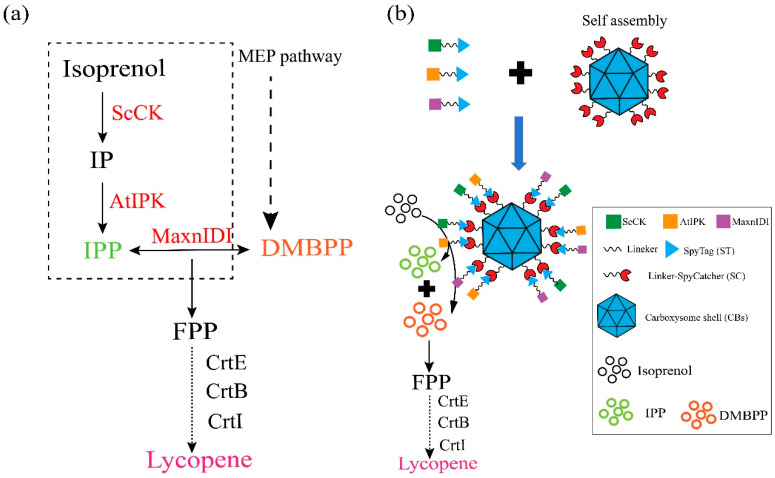
Schematic diagram of construction of IPP synthetic multi-enzyme nanocage for lycopene synthesis. (**a**) Enzyme cascade pathway for lycopene synthesis based on the isoprenoid alcohol pathway and endogenous MEP pathway. ScCK: choline kinase from *Saccharomyces cerevisiae*; AtIPK: isopentenyl phosphate kinase from *Arabidopsis thaliana*; MxanIDI: IPP isomerase from *Myxococcus xanthus*; IP: isopentenyl monophosphate; IPP: isoprenyl pyrophosphate; DMBPP: dimethylallyl pyrophosphate from the MEP pathway; FPP: farnesyl diphosphate. (**b**) Assembly of IPP synthetic enzyme on α-carboxysome shell using the SpyTag/SpyCatcher for lycopene synthesis.

The α-carboxysome simplified gene module from *P. marinus* MED4 was used for the assembly of carboxysome shells (CBs) (Supplementary Figure S1). To establish an IPP synthetic enzyme immobilized on carboxysome shells, we selected an exogenous IPP synthetic enzyme cascade based on the isoprenoid alcohol pathway [31,32,33,34]. This enzyme cascade involves the synthesis of isoprenyl pyrophosphate (IPP) from the exogenous addition of isoprenol by choline kinase from *Saccharomyces cerevisiae* (ScCK) and isopentenyl phosphate kinase from *Arabidopsis thaliana* (AtIPK). Subsequently, the interconversion of IPP and dimethylallyl pyrophosphate (DMBPP) is catalyzed by an IPP isomerase (IDI), accumulating IPP for the production of lycopene via the catalysis with CrtE, CrtB, and CrtI [44] (Figure 1a). The SpyTag/SpyCatcher (ST/SC) is a system for site-specific protein conjugation linked by intermolecular isopeptide bonds [45]. For the assembly of IPP biosynthetic enzymes on the exterior of the carboxysome protein cage, we fused the ST to the C-termini of ScCK, AtIPK, and MxanIDI with a (GGGGS)_2_ linker. The SC protein was then connected to the major carboxysome shell protein CsoS1A, which was also linked via a (GGGGS)_2_ sequence at its C-terminus (Figure 1b).

### 3.2. Expression and Characterization of Carboxysome Nanocage Scaffold in E. coli

**Figure 2:**
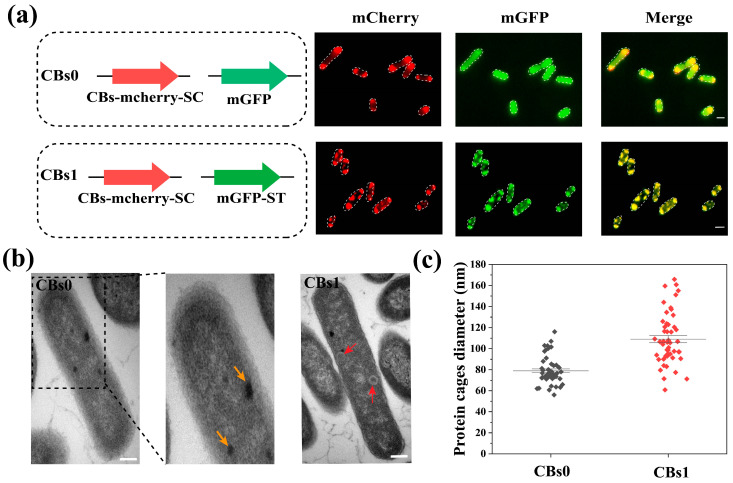
Characterization of the assembled nanocage scaffold in *E. coli*. (**a**) Schematic representation of the gene constructs for protein nanocage labeled with fluorescent proteins. CBs0, the *E. coli* strain expressing CBs-mCherry-SC and mGFP (control strain); CBs1, the *E. coli* strain expressing both CBs-mCherry-SC and mGFP-ST (**Left**). Confocal imaging of the engineered CBs1 and CBs0 (**Right**). Scale bar, 1 μm. (**b**) TEM images of engineered *E. coli* CBs0 and CBs1. For the CBs0, the nanocages are shown in the middle panel presenting an enlarged view of the area demarcated in the left panel. The nanocages are indicated with yellow arrows. For theCBs1, the nanocages carrying cargo proteins are indicated with red arrows. Scale bar, 500 nm. (**c**) Comparison of the average diameter of CBs in CBs1 with that in CBs0 (control strain) (*n* = 50).

First, we expressed the assembled CBs with green fluorescent protein (mGFP) as the cargo protein in *E. coli*. For visualization, we fused the red fluorescent protein (mCherry) with surface proteins of carboxysome shells. We co-expressed the ST-linked mGFP (mGFP-ST) and SC-linked CBs (CBs-mCherry-SC) in *E. coli* (strain CBs1) (Figure 2a). A strain (CBs0) expressing CBs-mcherry-SC and mGFP was used as the control. The mGFP-ST was co-localized with the SC-linked CBs in the cells compared with the control (Figure 2a), indicating that the cargo protein can be immobilized on the CBs. The intact spherical carboxysome shells in CBs0 and CBs1 were visualized by transmission electron microscopy (Figure 2b). The average diameter of the naked CBs-mcherry-SC in the control strain CBs0 was 79.5 nm, whereas the average diameter of the assembled nanocage in the CBs1 increased to 107.9 nm (Figure 2c), indicating that the cargo proteins were successfully attached to the surface of the protein cage. In addition, we found that the expression of CBs and the assembled protein cages had little effect on cell growth, suggesting that the protein cages are biocompatible with *E. coli* (Supplementary Figure S2).

### 3.3. Immobilization of IPP Synthetic Enzymes on Nanocages Led to Increased Lycopene Production in E. coli

**Figure 3:**
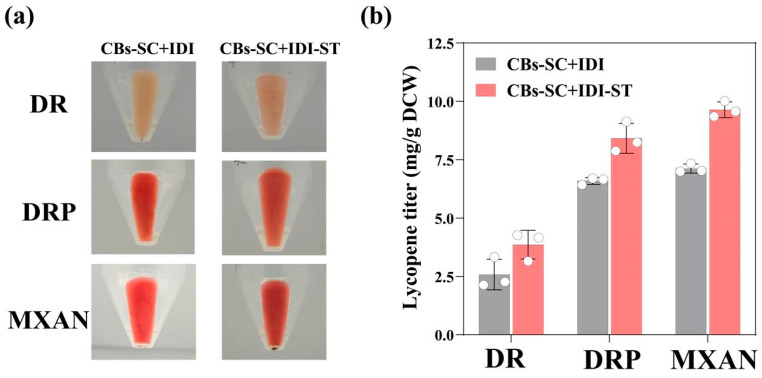
Immobilization of IDI on carboxysome nanocages improved lycopene production in *E. coli***.** (**a**) Comparison of cell pigment of *E. coli* ly001 strains co-expressed with CBs-SC+IDI or CBs-SC+IDI-ST, respectively. The IDI was from DR, DRP, or MXAN; (**b**) lycopene analysis of the *E. coli* strains using HPLC. Data are presented as mean ± standard deviation (*n* = 3).

To verify the functionality of enzyme immobilization on carboxysome nanocages, we first immobilized the IDI on the carboxysome nanocage, which is one of the rate-limiting enzymes for lycopene synthesis [46]. Exogenous IDI genes were screened in *Deinococcus radiodurans* (DR), *Deinococcus radiopugnans* (DRP), and *Myxococcus xanthus* (MXAN) based on bioinformatics analysis. Subsequently, we immobilized the IDIs on the nanocages by the conjugation of the C-terminal regions of IDI with ST-linker to the SC-linked CBs, which were expressed in the *E. coli* ly001 strain containing CrtE/CrtB/CrtI. All the strains were cultured to the OD_600_ of 0.8, and the inducer IPTG was added. After 48 h of overexpression at 25 °C, the cell cultures were centrifuged, and pellets were collected. As illustrated in Figure 3a, the strain with immobilized IDI on CB cages (CBs-SC+IDI-ST) exhibited a deeper red pigment compared to the strains with unimmobilized IDI (CBs-SC+IDI). Moreover, carotenoids were extracted from the cells, followed by HPLC analysis. Lycopene was identified as the major carotenoid product (Supplementary Figure S4), which was quantified against a standard calibration curve for lycopene (Supplementary Figures S3 and S4). The expression of IDI from MXAN (MxanIDI) or DRP (DrpIDI) in the strain CBs-SC+IDI-ST resulted in the higher yield of lycopene compared with the strains with unimmobilized IDI (CBs+IDI-ST), indicating that the strain with IDI immobilized on the CB nanocage could improve the lycopene production (Figure 3b). Therefore, the immobilization of rate-limiting enzymes within carboxysome nanocages is advantageous for product synthesis compared to free enzymes.

**Figure 4:**
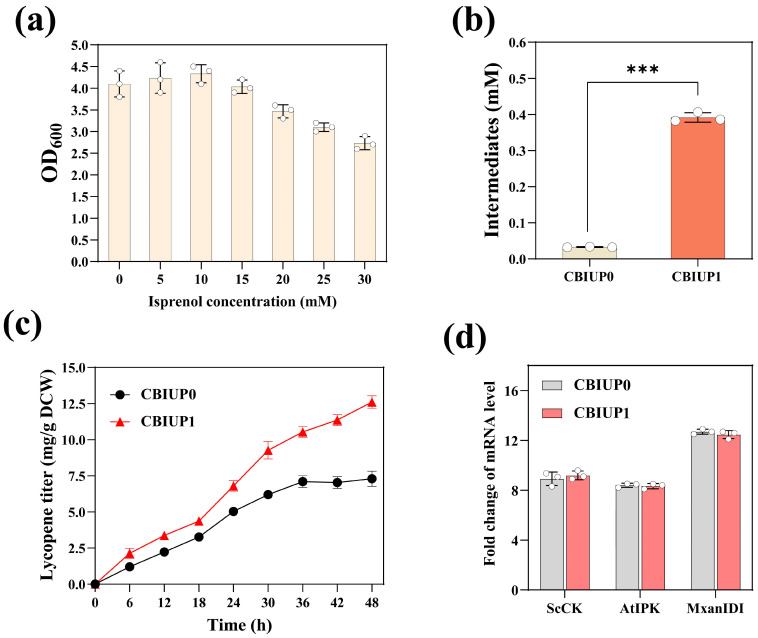
Assembled IPP enzymic on CB nanocages enhanced the lycopene production in *E. coli*. (**a**) Effect of isprenol concentration on *E. coli* growth. (**b**) IPP content in the CBIUP0 and CBIUP1 strains. ***, *p* < 0.001. (**c**) Lycopene synthesized in the CBIUP0 and CBIUP1 strains cultured for 48 h. Samples were taken at 6 h intervals to determine lycopene levels. (**d**) Relative mRNA levels of *ScCK*, *AtIPK*, and *MxanIDI* in the CBIUP1 compared to CBIUP0. Data are presented as mean ± standard deviation (*n* = 3).

Sufficient supply of IPP and DMAPP is crucial for the effective biosynthesis of terpenoids [47]. Furthermore, we immobilized the ScCK and AtIPK from the isoprenoid alcohol pathway for the IPP synthesis on the CB nanocages containing MxanIDI from MXAN. By fusing ST to the genes, we generated the fusion genes for expression of ScCK-ST and AtIPK-ST, respectively, resulting in IPP synthetic nanocages assembled with ScCK, AtIPK and MxanIDI. Then, we constructed a plasmid containing the three genes involved in IPP (isopentenyl pyrophosphate) synthesis and co-transformed it with CBs-SC into the ly001 strain to enhance lycopene biosynthesis. Two strains were constructed: CBIUP_1_, which contained multi-enzymic IPP synthetic cages, and the control strain CBIUP_0_, which contained only “free” enzymes. The CBIUP1 strain expresses the ST-linked enzymes and CBs-SC, facilitating the spontaneous attachment of the enzymes to the exterior of the CB protein cage, resulting in the assembly of the ScCK/AtIPK/MxanIDI-CBs (Figure 1b). The distinction between CBIUP_1_ and CBIUP_0_ is the absence of ST in ScCK, AtIPK, and MxanIDI. The addition of isoprenol might influence the growth and metabolism of the cells [48]. However, it was observed that the addition of 15 mM isoprenol resulted in little impact on the CBIUP0 strain’s growth (Figure 4a). Additionally, we compared the lycopene yield of CBIUP1 and CBIUP0 during fermentation. After 48 h of fermentation, the CBIUP1 achieved a lycopene production of 12.6 mg/g DCW for strain, which is 1.7 times greater than that of CBIUP0 (7.3 mg/g DCW) (Figure 4c). We monitored the levels of metabolites involved in the biosynthesis of lycopene. Notably, the IPP levels in the CBIUP1 strain increased about 11.8-fold compared with the CBIUP0 after 24 h of induction (Figure 4b). There were no significant changes in the transcription level of the relevant biosynthetic genes, which could eliminate the possibility that variations in expression level of enzymes contributed to differences in lycopene production (Figure 4d). Therefore, the increase in lycopene yield can be attributed to the assembly of IPP synthetic enzymes on the nanocages. The assembled IPP synthetic CB cages might accumulate related enzymes and enhance metabolic flux for lycopene production. The enzymes might be attached spontaneously to the exterior of the CB nanocages through the SC-ST system. The detail and ratio of binding for the ScCK/AtIPK/MxanIDI to CB nanocages need to be investigated in future study.

## 4. Conclusions

In this study, we developed a strategy for organizing IPP synthetic enzymes on carboxysome protein cages to enhance the lycopene synthesis. By employing ST/SC methods, we successfully achieved attachment of the IPP synthetic enzymes (ScCK-AtIPK-MaxnIDI) to the surface of protein cages and expressed them in *E. coli*. The engineered strain expressing the self-assembling IPP synthetic nanocages demonstrated a 1.7-fold increase in lycopene production compared to the control strain expressing free enzymes. This work provides evidence supporting that the CBs can serve as an ideal scaffold for constructing customized multi-enzyme assemblies, which have broad applications in synthetic biology and metabolic engineering.

## Data Availability

The original contributions presented in this study are included in the article/Supplementary Materials. Further inquiries can be directed to the corresponding authors.

## Supplementary Materials

The following supporting information can be downloaded at https://www.mdpi.com/article/10.3390/microorganisms13040747/s1. Figure S1: Gene modules of the carboxysome from *P. marinus* MED4 utilized in the constructed expression plasmids. Figure S2: Comparison of cell growth between engineered strains by monitoring OD_600_. empty vector: Strain ly001 was transformed with the empty vector pACYCDuet-1 and PCDFDuet-1. Data are presented as mean ± standard deviation (*n* = 3). Figure S3: The calibration curve for lycopene analysis using HPLC. Figure S4: HPLC analysis image of IDI immobilized on carboxysome nanocages. Comparison of cell pigment of *E. coli* strains co-expressed IDI/IDI-ST with CBs-SC, respectively. The IDI was from DR, DRP or MXAN. Table S1: Primers used in this study. Table S2: Plasmids used in this study. Table S3: Strains used in this study. Table S4: The nucleotide sequence of the gene in this study.
