# Supplementary material for: Enhancement of Lycopene Biosynthesis Using Self-Assembled Multi-Enzymic Protein Cages
Source: Microorganisms. 2025 Mar 26;13(4):747. doi: 10.3390/microorganisms13040747 (PMC12029616; doi:10.3390/microorganisms13040747)
Supplement: Supplementary file 1 [file microorganisms-13-00747-s001.zip › microorganisms-3511387-supplementary.pdf]

Enhancement of lycopene biosynthesis using self-assembled multi-enzymic protein cages

Yulong Zhou[1,2], Yonghua Yao[1,2] , Furong Zhang[2], Ning Yu[2], Binqiang Wang[2 *], Bing Tian [1,2*]

1. Key Laboratory for Green Processing of Chemical Engineering of Xinjiang Bingtuan, School of Chemistry and Chemical Engineering, Shihezi University, Shihezi 832003, China.
2. Institute of Biophysics, College of Life Sciences, Zhejiang University, Hangzhou 310058, China.

*Correspondence: wangbinqiang@zju.edu.cn; tianbing@zju.edu.cn

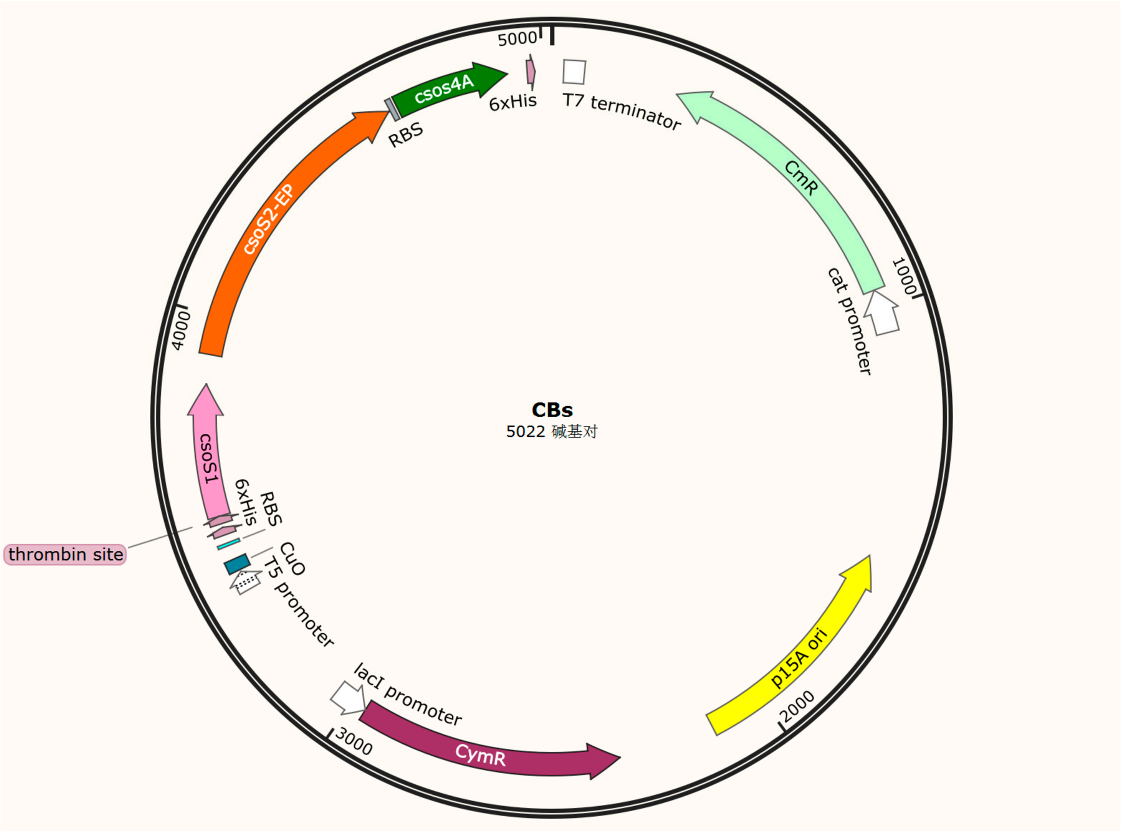

Supplementary Figure S1. Gene modules of the carboxysome from *P. marinus* MED4 utilized in the constructed expression plasmids.

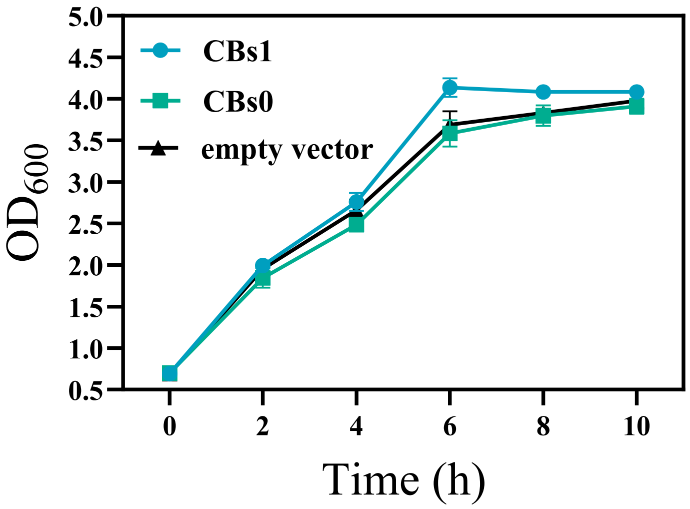

Supplementary Figure S2. Comparison of cell growth between engineered strains by monitoring OD_{600}. empty vector: strain ly001 was transformed with the empty vector pACYCDuet-1 and PCDFDuet-1. Data are presented as mean ± standard deviation (n=3).

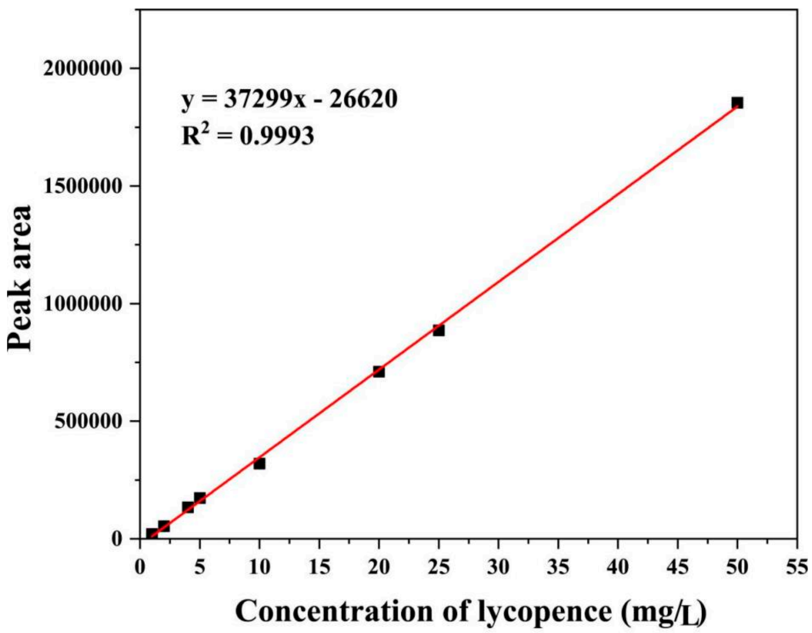

Supplementary Figure S3. The calibration curve for lycopene analysis using HPLC.

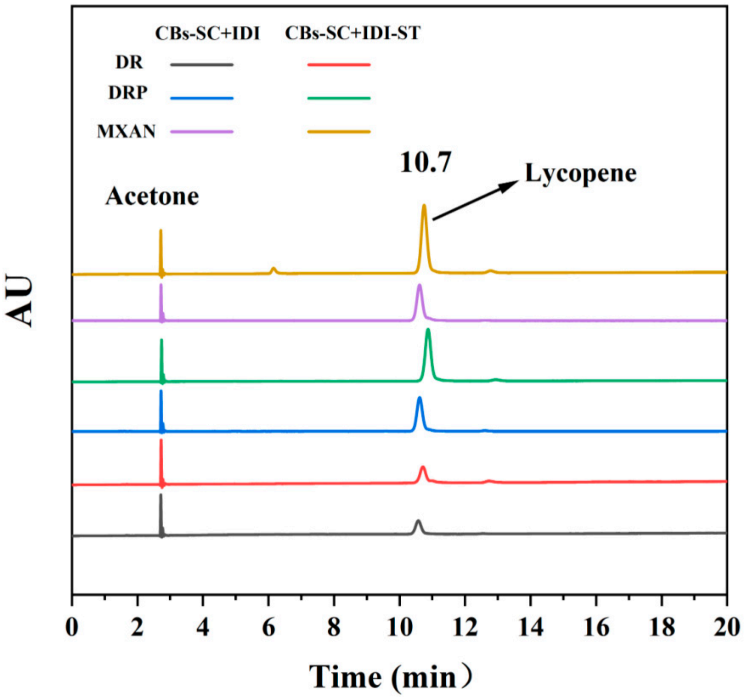

Supplementary Figure S4. HPLC analysis of carotenoid extracts from *E. coli* ly001 strains co-expressed with CBs-SC+IDI or CBs-SC+ IDI-ST, respectively. The IDI was from *Deinococcus radiodurans* (DR), *Deinococcus radiopugnans* (DRP) or *Myxococcus xanthus* (MXAN).

Supplementary Table S1. Primers used in this study

Name	Sequence (5`-3`)<br>The lowercase letter are homologous arms
CBs-mCherry-SC-F	acgagctgtacaagggatCAGGTGGAAGTATGGGTAGCAGCGATAGCGC
CBs-mCherry-SC-R	ttaatttacttgtacagctcTTAAATATGGGCATCACCTTTGGTTGC
mGFP-F	gtataagaaggagatatacatATGGTGAGCAAGGGCGAGGAG
mGFP-R	aattaagctgcgctagtagaTTACTTGTACAGCTCGTCCATGCCGAGAGT
mGFP-ST-F	ttatggttgatgcatataaacCGACGAAATAATCTACTAGCGCAGCTTAATTA
mGFP-ST-R	gtttatatgcatcaaccataaCAATATGTGCCTTGTACAGCTCGTCCATGCCG
Pp15ACB-F	TCTACTAGCGCAGCTTAATTAACCTAGGCTG
Pp15ACB-R	ATGTATATCTCCTTCTTATACTTAA
CBs-SC-F	ggcggtagtggtggttcaggTGGTAGTATGGGTAGCAGCGATAGCGC
CBs-SC-R	tagtattctcctctttaatTTAAATATGGGCATCACCTTTGGTTGC
MXAN-IDI-ST-F1	cggtggtggtagttctactATGGGCGACGACATCACTG
MXAN-IDI-ST-R1	CAGCGCCGCCAACCAATC
MXAN-IDI-ST-F2	gattggttggcggcgctgGGTGGTGGTGGTAGTGGTG
MXAN-IDI-ST-R2	tcgaattcggatcctggctTTATTTCGTCGGTTTATATGCATCAACCA
MXAN-IDI-R	tcgaattcggatcctggctTTACAGCGCCGCCAACCAATC
DR-IDI-ST-F	cggtggtggtagttctactATGTCGGGCGTGAGACT
DR-IDI-ST-R	ccaccactaccaccaccaccCGCGCCCACCCCGG
DR-IDI-R	ctcgaattcggatcctggctTTACGCGCCCACCCCGG
DRP-IDI-ST-F	cggtggtggtagttctactATGACCCTGGTCCGGC
DRP-IDI-ST-R	ccaccactaccaccaccaccGCCGGACTCGGTGGCCG

DRP-IDIR	ctcgaattcggatcctggctTTAGCCGGACTCGGTGGCCG
ScCK-AtIPK-IDI-F	tcgatactagttatttgtaaATAAGTGATAATAAGCGGATGAATGGCAGAAAT
ScCK-AtIPK-IDI-R	aattaagctgcgctagtagaTTACAGCGCCGCCAACCAAT
[ScK-AtIPK-IDI]-ST-F	acgtcgacaagcttgcataaATAAGTGATAATAAGCGGATGAATGGCAGAAAT
[ScCK-AtIPK-IDI]-ST-R	aattaagctgcgctagtagaCTGCAGGCGCGCCGA
CrtE-F	gccgcgcggcagccatatgATGCGTCCTGATCTGCTGTCCC
CrtE-R	ctagtattctcctctttaatTCAGGCGTCGCGCGT
CrtB-F	attaaagaggagaatactagATGATCAACACGTCCCTGCCTC
CrtB-R	ctagtactttcctgtgtgaTTACCCACTGACCGCGCC
CrtI-F	tcacacaggaaagtactagATGACTTCCCCTTCTCTGAATACCAG
CrtI-F	cggagctcgaattcggatccTCAGTCGGTTGCGCCCG
PET28a-F	GGATCCGAATTCGAGCTCCGT
PET28a-R	CATATGGCTGCCGCGCG

Supplementary Table S2. Plasmids used in this study

Plasmids	Description	Source
pET28a-CrtE/B/I	pET28a ligated with *crtE*, *crtB* and *crtI*, kan+	This study
pP15A-CBs	pACYCDuet-1 ligated with *csoS1A*, *csoS2-ep* , *csoS4A*, Cm+	This study
pP15A-CBs-SC	pACYCDuet-1 ligated with *csoS1A-spycatcher, csoS2-ep, csoS4A* , Cm+	This study
pP15A-CBs-mCherry-SC	pACYCDuet-1 ligated with *csoS1A-mcherry-sptcatcher, csoS2-ep, csoS4A* , Cm+	This study
PCDF-mGFP	PCDFDuet-1 ligated with *mgfp*, Str+	This study
PCDF-mGFP-ST	PCDFDuet-1 ligated with *mgfp-st* , Str+	This study
PCDF-DrIDI	PCDFDuet-1 ligated with *dridi*, Str+	This study
PCDF-DrIDI-ST	PCDFDuet-1 ligated with *dridi-st* , Str+	This study
PCDF-DrpIDI	PCDFDuet-1 ligated with *drpidi* , Str+	This study
PCDF-DrpIDI-ST	PCDFDuet-1 ligated with *drpidi-st* , Str+	This study
PCDF-MxanIDI	PCDFDuet-1 ligated with *mxanidi* , Str+	This study
PCDF-MxanIDI-ST	PCDFDuet-1 ligated with *mxanidi-st* , Str+	This study
PCDF-ScCK-AtIPK-MxanIDI	PCDFDuet-1 ligated with *scck* , *atipk* ,and *mxanidi* , Str+	This study
PCDF-[ScCK-AtIPK-MxanIDI]-ST	PCDFDuet-1 ligated with *scck-st* , *atipk-st* ,and *mxanidi-st* , Str+	This study

Supplementary Table S3. Strains used in this study

Strains	Description	Source
ly001	*E.coli* BL21(DE3) harboring pet28a-CrtE/B/I	This study
CBs0	*E.coli* BL21(DE3) harboring pet28a-CrtE/B/I, pP15A-CBs-mCherry-SC and PCDF-mGFP	This study
CBs1	*E.coli* BL21(DE3) harboring pet28a-CrtE/B/I, pP15A-CBs-mCherry-SC and PCDF-mGFP-ST	This study
CBIUP0	*E.coli* BL21(DE3) harboring pet28a-CrtE/B/I, pP15A-CBs-SC and PCDF-ScCK-AtIPK-MxanIDI	This study
CBIUP1	*E.coli* BL21(DE3) harboring pet28a-CrtE/B/I, pP15A-CBs-SC and PCDF-[ScCK-AtIPK-MxanIDI]-ST	This study

Supplementary Table S4. The nucleotide sequence of the gene in this study

Gene	Nucleotide sequence
csoS1A	atggctacagaaacaatgggtatcgcactcggcatgatcgagacacgcggacttgtacctgcaatc gaagcagcagacgcaatgaccaaggcagcagaagttcgtcttattggtcgtgaattcgtaggtggc ggttatgtcacagttttggttagaggagaaacaggagctgtcaacgcagcagtaagagctggagct gatgcttgtgagagagttggtgatggtctcgtcgcagctcacatcattgctcgccctcatagagaagt tgagccagcacttggtaatggcgatttccttggtcaaaaggactaa
csoS2-EP	atgaaatctcgagcgacagtcaattctggcaataattcaaatgctagattaactggactgcagccag gcattggtggagtaatgactggagcaacaaaaggttcttgtaaaaatttaactggaaccccatatatt ggaggagatcagtttttatcaaattgcgaaacaccaccaaacgatgcttcttatgcaaatcaagagaa gtcagcctcaaattcttggaaggaattttctgttaattcaccatcaagagaaaagtattcagctaaaaat acagaaggagtaacaggtaataggtatgaagatagttcaaagattacaggaccttttgatatggcag aagataaagtcacgggtactgagcaatttagatttgaacctaataaaaatatgacttataaacaaaaa atgaaacaagaagaaagtcaaaatattgatattcctacagataaaaaagagccttctaaaataacag

	gtgagggtcaatctgcagggaatataacaggtgatgactgggatagaggagataaagttacggga<br>acagaaggagtttctgctaggaagaggaatccatcaagagcgggatttatgggggcaatgccacc<br>tgttgataataagagaaatgatgagacagaaaaacctgattttcttataactggatctagtggtaacac<br>tcgcgatggacaactggttaccttttcaggtggtgcaagaggttaa
csoS4A	atgcttatttgcaaggtattgaaaccacttgtttcaacaaatagaataccgggattcgagcataagcat<br>cttcaagtagttttagatggctcttctaataaggttgccgttgatgctgtgggatgtaagcccggtgatt<br>gggttatttgtgttggtagttctgctgctagagaagctgcagggagtaagtcatatccaagtgatttaa<br>cgattgttggaattattgatcattgggatcccgatagtccaaaacagattgaggtgtag
mcherry	atggtgagcaagggcgaggaggataacatggccatcatcaaggagttcatgcgcttcaaggtgca<br>catggagggctccgtgaacggccacgagttcgagatcgagggcgagggcgagggccgcccta<br>cgagggcacccagaccgccaagctgaaggtgaccaagggtggccccctgcccttcgcctggga<br>catcctgtcccctcagttcatgtacggctccaaggcctacgtgaagcaccccgccgacatccccga<br>ctacttgaagctgtccttccccgagggcttcaagtgggagcgcgtgatgaacttcgaggacggcgg<br>cgtggtgaccgtgacccaggactcctccctgcaggacggcgagttcatctacaaggtgaagctgc<br>gcggcaccaacttcccctccgacggccccgtaatgcagaagaagaccatgggctgggaggcctc<br>ctccgagcggatgtaccccgaggacggcgccctgaagggcgagatcaagcagaggctgaagct<br>gaaggacggcggccactacgacgctgaggtcaagaccacctacaaggccaagaagcccgtgca<br>gctgcccggcgcctacaacgtcaacatcaagttggacatcacctcccacaacgaggactacacca<br>tcgtggaacagtacgaacgcgccgagggccgccactccaccggcggcatggacgagctgtaca<br>agtaa
spycatcher	atgggtagcagcgatagcgcaacgcatattaaatttagcaaacgtgatgaagatggcaaagaactg<br>gcaggtgcaacgatggaactgcgtgatagcagcggtaaaacgattagcacctggattagcgatgg<br>tcaggtaaaagatttttatctgtatccaggtaaatatacctttgttgaaaccgcagcaccggatggctat<br>gaagttgcaacggcaattacctttaccgttaacgaacagggtcaggttaccgttaatggtaaagcaa<br>ccaaaggtgatgcccatatttaataa
mgfp	atggtgagcaagggcgaggagctgttcaccggggtggtgcccatcctggtcgagctggacggcg<br>acgtaaacggccacaagttcagcgtgtccggcgagggcgagggcgatgccacctacggcaagc<br>tgaccctgaagttcatctgcaccaccggcaagctgcccgtgccctggcccaccctcgtgaccaccc<br>tgacctacggcgtgcagtgcttcagccgctaccccgaccacatgaagcagcacgacttcttcaagt<br>ccgccatgcccgaaggctacgtccaggagcgcaccatcttcttcaaggacgacggcaactacaag<br>acccgcgccgaggtgaagttcgagggcgacaccctggtgaaccgcatcgagctgaagggcatc<br>gacttcaaggaggacggcaacatcctggggcacaagctggagtacaactacaacagccacaacg<br>tctatatcatggccgacaagcagaagaacggcatcaaggtgaacttcaagatccgccacaacatcg<br>aggacggcagcgtgcagctcgccgaccactaccagcagaacacccccatcggcgacggcccc<br>gtgctgctgcccgacaaccactacctgagcacccagtccgccctgagcaaagaccccaacgaga<br>agcgcgatcacatggtcctgctggagttcgtgaccgccgccgggatcactctcggcatggacgag<br>ctgtacaagtaa
(GGGGS)2	ggtggtggtggtagtggtggtggtggttca
drpcrtE	atgcgtcctgatctgctgtcccgcgtgctgtcgctgctgcccgagtccgggacgcgccccgaatac<br>cagcagtaccacgacatgctgcgcgactacccccggcgcggcggcaagggcattcgcagcgaa<br>ctgctgctcgccagcgcccgcgcgcacggcgtgaccgtggacacgccgcagtgggaggccgc

	gttgtggctggccgccgcgctggagctgttccagaactgggtgctgatccacgacgacatcgagg<br>acgactccgaggaacgccggggcaaaccggccctgcaccgcctgcacggcgtgccgctggcc<br>atcaacgccggggacgccctgcacgcctacatgtgggccgccgtccaccgcgccgacgtgccc<br>ggcaccatgcccgaattcctgaacatgatccaccacaccgcccagggccagcacctggacttgtg<br>ctgggtggagggccgccgctgggatctgatggaggacgactacctggaaatggtgcgccagaag<br>accgcgtactacacggtggtcattccgctgcggctgggggcgctggcggcgggtgttcagccatc<br>cgagcagttcacgcccgctggcctggcgctgggcgcggccttccagatccgtgacgacgtgctg<br>aacctggcgggcgacgcggccaggtacggcaaggaaatcggcggcgatctgctggagggcaa<br>gcgcaccatgatcgtgctgcactggctgcgcaccgcgccggcagcccagcgccaggccttcctg<br>aagcagatgggcctgaaccgccccgacaaggacgcccggaccattgccgacattcaccgctggt<br>tgctggacagcggcagcgtcacctacgcccaggagtacgcccacgcccaggccgagcagggg<br>ctggcgctgctttcggaggcgttcgcggaggctccaggccagcaagcagcggcggaactgctgg<br>ccgccatgcgcgaactcgcgacgcgcgacgcctga
drpcrtB	atgatcaacacgtccctgcctccctccccgacttccgctgccccggtgcaggcggtggcccattgc<br>cgggacgtgacgcgggaccacagcaagaccttctttctgggctcgcggctgtttccgctgcggca<br>gcggcaggcggtctgggcggtgtacgcggcctgccgcaccggggacgatattgccgacgagtc<br>caccgccgacagcgtcgaggccgaactcgacgtgtggtggacccggattcagggggcttttgcc<br>gggcgccccggccccgatcccgtggacacggcgctggcctgggctgcccgcacctacccgattc<br>cgctgtcggcctttgccgaattgcacgagggcctgcggatggacctgaacggccacgtctaccac<br>gacatggaggatctggcgctgtactgccgccgggtggccggcgtgatcggattcatgatcgcgcc<br>ggtcagcgggtacagcggcggcgagcgcaccctgcaccacgccctgatgctggggcaggccat<br>gcaactgaccaacatcctgcgcgacgtgggcgaggacctgacgcgtgggcgggtctacctgccc<br>cagaccctgctgggggagtacggcgtgagccgcgccgacctggagcgcggcgtggttacgccc<br>gagtaccgcgccctgatggttcacctgtgtgccctggcccgcgactggtacgccgagggccgcg<br>ccgggattccctgcctgcacggcagcgcccggctggccgtcgccaccgccgcccgcgcctacg<br>agggcattctggacgatctggaacgcgccgactacgacaacttcaaccgccgcgcctatgtcagc<br>ggcacccgcaaactgctgatgctgccccgcgcgtggtgggaactgcgcggcgcggtcagtgggt<br>aa
drpcrtI	atgacttccccttctctgaataccagcaggcgcaagaccgccctcatcatcggctcgggcatcggc<br>gggctgtcgctgggcatccggctgcaaagcctgggcttcgacaccaccatcttggaacgcctgga<br>cgcgccgggggggccgcgcgtaccagaagcgcaccgaggacggctacgtgttcgacatggggc<br>cgacggtgatcacggtgccgcattttatcgaggaactgtttgcgctggaacgcgacaagggcatgc<br>tgggcgagccggactacccgccccacacgctgaccgaggacgcccgcgtcaagactggcgag<br>agcggcggcccgcgcacccgcgagtacgtgaaactggtgccgatcctgcccttttaccggatttat<br>ttcgacgacggctccttcttcgactacgacggcgatcccgaaagcacccgccgtcagatcggcga<br>actcgcgccggaagacctgaccgggtacgagcgcttccacgccgacgcccgcgccatcttcgag<br>cgcggctttctggaactgggctacacccactttggcgacatgaccagcatgctgcgcgtcgtgccg<br>gacctgatgcgcctggacgccgttcgcacgctgttctcgtttaccagcaagtatttccagaacccca<br>agatgcggcaggtcttttccttcgagacgctgctggtgggcggcaacccgctgagcgtgccggcc<br>atctacgccatgatccacttcgtcgaaaaaacctggggcatccactacgcgatgggggggcaccgg<br>ggcgctggtgcgcgccttcgtgcggaagttcgaggaactgggcggcacgctgcgcctgaacgcg

	ggcgtggacgagattctggtcacggacgggcgcggccagccggtcaggcatccagtcggcaag<br>cggacggcgcgcggggtgcggctggagggcggcgaggaactgcacgccgacatcgtggtcag<br>caacggcgactgggccaacaccaacctgaggcgcattcccgccgccgcccggctggtcaacag<br>cgacatgcgcgtgaaggccgcccgccagagcatgagcctgctggtgatctacttcggcttccgcg<br>atgatccggcccgcccgctggacctgcgccaccacaacatcatcctggggccgcgctacgagga<br>actgctcaccgagattttcgggcagaaggtgctgggccaggatttcagccagtacctgcatgtgcc<br>gacgctgaccgatcccagccttgcccccgccgggcatcacgccgcctacaccctggtgccggtc<br>ccgcacaacgccagcggcctggactgggcggtggaggggccgaagctggtggaccgcgtgta<br>cagcttcctggaggaacgcggctacatcccgaacctgcgcgagcgcctgacccacagcgagtac<br>atcacccccgattacttcgcccagacgctggacagctacctgggcaacgcctttggccccgaaccc<br>gtgctggttcagagcgcgtacttccgcccgcacaaccgctcggaggacatcggcaacctgtacttg<br>gtgggtgcgggcgcgcagcccggcggtggcacccccagcgtgatgatgtcggccaagatgacg<br>gcccgcctgatcgccgatgactttgggattcaccgggccgtgcgggacggcgtgcccgccgccg<br>ctgaagcactggtgagggaagtggcgggcgcaaccgactga
drpidi	atgaccctggtccggcccccgacatcgcggcgcgcaaactgcgccatgtggacgcctgcctgct<br>gcccgaaagccagtacgcgggcgtgaccaccgggctggaggccgtgccctggccctaccgcgc<br>cctgcccgagcgcaacctgagcgacgtcgacctggggaccactttcctgggccgccgcctgagc<br>gccccgctgctgatcggcgcgatgaccggcggggccgagcgcgccgggcgcatcaacgccca<br>tctggcgcgggcggcgcagaacctgggcatcgggctgatgctgggctcgcagcgcgtcatgctg<br>gagcgccccgacaccgccgccacctttcaggtgcgtgaactggcccccgacatcctgctcgtggg<br>caatctgggcggggcgcagttcctgctgggctacggcgcgaaggaagccgtgcgcgccgtgcg<br>gacggtgggggccgaagccctggcgatccacgtcaacccccctgcaggaagcgttgcaaagtgg<br>cggcgacaccaactgggccggattgaccgcgcggctggccgaactgctgccccaattgccctttc<br>cggtgattcttaaagaggtggggcatgggctggacgcgaggacggtggccgccgtgtcgggtct<br>gggctttgccgcgctggacgtggccggggccggggggaccagctgggcgcgcgtggagcaac<br>tggtggaacgcggcgcggtcatcagccctgacctgtgcgaggtgggcgtgccgacggcgcagg<br>ccctgcgcggtgccagggccgccgcgccgcagacgccgctgatcgcctcgggggcatccgg<br>accgggctggacgccgcccgtgccctgctgctgggcgcgcaggtggtggcggtggcccgtccg<br>ctgctcgcccccgcgctcgacagcgccgaggccgccgaggactggctggcgaactttatccacg<br>agttgcgcgtggccctgttcgtgggggggctacggggacgtgtcggcggtgcgggccgccggcg<br>cgtcatccagggcgtcggccaccgagtccggctaa
spy-tag (ST)	gcacatattgttatggttgatgcatataaaccgacgaaataa
dridi	atgtcgggcgtgagactcaccgttctgggcagtacaggcagcatcggcacgcaaacactggacgt<br>ggcgcggcagcgcgggtatacggtgggcacgctcgccgccggacgcaatctggacttgctcgc<br>cgagcaggtgcgcgagttccggcccgccctggtcagcgtggacgggtccattctggccgaggcg<br>agagaacggctgagcggagtgcgcgtgattgccgacccctccgaagcagcggtggcacaggcc<br>gatgtggttgtcaacgccatgagcggcctgattggtttgccccccacccgcgccgcactcgaagcc<br>gggcaggcggtggcactggcgacgaaggaagcgatggtcacgtcggcccacttgatgtgggaa<br>gcggcggcagcgggtgggggccgggtggtgccggtagattccgagcacaccggggtctacca<br>gtgcctgaccggcgaggatatgggggacgtggccgagttgattctcacggcatcgggcgggccg<br>tttcgggatggccccgccgacctgagcgcggtcacgcctgagcaggcgctgaagcatccgtcgt

	ggagcatggggcccaaagtgaccatcgactcggccaccctgatgaacaagggcctggaagtcat<br>ggagtgtgccagcctctacggcctgccgctctcgcaggtgggcgtggtcgttcatccgcagagcat<br>cgttcacgcggcggtgcgattgcgcgacggcagcctgaaagcgcagttcggcccagcggacatg<br>cgcctgccgattgcctacgccatcgacgccgcgccgagcgggatgcaacaccccggcgacgtg<br>cgcggggcgcggcgtggggaagaggtcggggagcatctcggctggccgctgctggggcagtg<br>ggaattccgggcaccggacctgacgcgctttccctgcctcgcgctcgcttaccgggcgggtgagg<br>cgggcggactcctgccagtggcgctcaacgcggcggacgaggtggcggtggacgctttcctgca<br>cgggcgcatcggctttctggacattccccagctgattgaaagggtgctggacgagacgcccggcg<br>gcgccctcacctgggacagcctgacggagacggacgcctgggcacgcgccagagcgcaggaa<br>ctctgtgcctacggggtcggcggcgccggggtgggcgcgtaa
maxnidi	atgggcgacgacatcactgccagacgcaaggacgcgcatctcgacctttgctcgacgggggacg<br>tcgaacccagcggaaacagcaccctgctggagtgcgtcaagctggtccactgcgcgatgccgga<br>aatgtccgtggaggacgtggacctgtccacggccttcctgggcaagcggctgcgctacccgctgc<br>tcgtcaccggcatgacgggtgggacggagcgtgcgggtgcggtgaatcgcgacctggcgctgct<br>cgccgagcgccacggcctggccttcggcgtgggcagccagcgcgccatgtcggaggacgcctc<br>gcgggccgcgtccttccaggtgcggcaggtggcgcccacggtggcgctcctgggcaacatcgg<br>catgttccaggccatcgggctgggcgtggatgggacgcgccggctggtggacggcattggcgcg<br>gacgggctggcgctgcacctcaacgcgggccaggagctgacgcagccggaaggcgaccggg<br>acttccagggcggctaccgcgtggtggagctgctggtgaaggcctttggcgaccggctgctggtg<br>aaggagacgggctgcggcattggcccggacgtcgcgcggcggttggtggacctgggcgtgcgg<br>aacatcgacgtgtccggtctgggcgggacgtcctgggtgcgcgtggaacaacttcgcgcgtcgg<br>gcgtacaggcacagttgggggcggagttcagcgcgtggggcattcccacggcggcggcgttgg<br>cctccgtgcgccgggccgtgggcccggacgtccacctggtggcgagcggtggcctgcgcacgg<br>ggctggacgcggccaaggtgctggcgctgggggcgaacctggctggcatggcgctgccgttgtt<br>ccgggcgcagcaggcgggtgggctcgaggcggcggaggcggcgctggaggtcatcctggcg<br>agtctgcggcaggcgctcgtgctgacgggaagcagaagctgcgctgaactgagacagcggccc<br>cgggtggtcaccggagagttgaaggattggttggcggcgctgtaa
scck	atggtacaagaatcacgtccagggagtgtaagaagttactcggtcggttaccaagcaaggtccaga<br>tcgagttctcaaagaagacattcgttaacacgccaacgttcctcgcaaagactgattagaaccatca<br>gtatcgagtctgatgtgtctaatattactgacgatgacgatttgagagctgtcaatgagggagtagcg<br>ggtgtgcaactggacgtctctgaaaccgcaaataagggaccaagaagagcatcagcaactgatgt<br>cacagatagtttgggttcgacttcgtcggaatatattgagattccctttgttaaggaaacattggatgca<br>agtttaccttcggattatctgaagcaggacatattaaatctcattcagagtttgaagatatccaaatggt<br>ataacaacaagaaaatccaaccggtagcacaagatatgaacttagtcaagatctctggtgcgatga<br>caaacgcaattttcaaagttgaataccctaagttaccatcgttgctattgagaatatacggaccgaata<br>ttgataatatcattgacagggaatatgaattgcagattttggctaggctttcattgaaaaatataggtcct<br>tccctttacggctgttttgtaaacggtagatttgagcagtttctggagaattctaagactttaacaaaag<br>acgacattagaaactggaagaactctcaaaggattgcaaggagaatgaaggagttacatgtaggtg<br>ttcctctcttgagttcagaaaggaagaacgggtcggcttgttggcaaaagattaaccagtggttgcg<br>cacgattgagaaagtcgaccaatgggtgggggatcctaaaaacattgaaaactctttattatgtgag<br>aattggtccaagtttatggatattgtcgatagatatcacaagtggcttatttctcaagaacagggtatag

	agcaagtcaacaaaaatcttatattctgccataatgatgcccaatacggcaatttacttttcactgctcc<br>tgtgatgaacacaccgagcctatacactgcaccttcgtctacatcattgacttcccaatcaagttcctta<br>tttccttcgagctccaatgtcattgtagatgatataatcaacccgccaaagcaggagcaaagccaag<br>attccaaattggtcgtcattgattttgaatatgcaggtgccaatcccgccgcatatgatttagcgaatca<br>tctttccgagtggatgtatgattacaacaatgctaaggccccacatcagtgccacgctgatagatatc<br>ccgataaagaacaggttttgaatttcttatactcttatgtttcgcatctaaggggtggtgctaaggaacc<br>catagatgaagaggttcaaagactctataagtcaatcattcaatggagacccactgtacaactattttg<br>gtcgctctgggccatcctacaaagtggtaaattagagaaaaaagaagcctccactgccatcactag<br>agaagaaattggacccaatggaaaaaaatatatcatcaagactgaacccgaatcccctgaagaag<br>actttgttgaaaatgacgacgagcctgaagctggcgtcagcattgacacgttcgattatatggcttat<br>ggtcgtgacaagattgcggtcttttggggcgacctcattggcttaggcataatcaccgaagaagaat<br>gcaaaaatttcagctctttcaagttcctcgatactagttatttgtaa
atipk	atggaactgaacatctctgaatctcgttctcgttccatccgttgcatcgttaaactgggtggtgcagct<br>atcacctgcaaaaacgaactggagaagatccacgatgagaacctggaagttgttgcttgccagctg<br>cgtcaggctatgctggaaggttctgcgccatccaaagttatcggtatggactggagcaaacgtccg<br>ggttcttccgaaatctcttgcgatgtagacgacatcggtgaccagaaatcttccgagttcagcaaatt<br>cgttgttgttcacggtgctggttctttcggccacttccaggcaagccgttctggtgttcacaaaggtgg<br>tctggagaaaccaatcgttaaagcgggtttcgtggctactcgtatcagcgttaccaacctgaacctg<br>gaaatcgttcgtgcactggctcgtgaaggtattccgaccatcggtatgtctccgtttagctgcggttg<br>gagcacctccaaacgtgacgttgcatctgctgatctggctaccgttgcaaagaccatcgattccggc<br>ttcgttccggtactgcacggtgacgctgttctggataacatcttgggttgcaccattctgtctggtgac<br>gtgatcatccgtcacctggcagaccacctgaaaccggaatacgttgtgtttctgactgacgttctggg<br>tgtatacgaccgtccaccatctccgtccgaaccggatgcagtgctgctgaaagaaatcgcagttggt<br>gaagatggctcttggaaagttgttaacccgttgctggaacacaccgacaagaaagtggactactctg<br>ttgctgcacacgacactactggtggtatggaaaccaagatctccgaagcagctatgatcgctaaact<br>gggcgtagatgtgtacatcgtcaaagcggcgaccactcactctcagcgtgcgttgaacggtgatct<br>gcgtgattctgtaccagaggattggctgggtactatcatccgtttctctaagtaa
